# Exploring Stress and Problematic Use of Short-Form Video Applications among Middle-Aged Chinese Adults: The Mediating Roles of Duration of Use and Flow Experience

**DOI:** 10.3390/ijerph19010132

**Published:** 2021-12-23

**Authors:** Qing Huang, Mingxin Hu, Hongliang Chen

**Affiliations:** College of Media and International Culture, Zhejiang University, Hangzhou 310058, China; qing_huang@zju.edu.cn (Q.H.); 12123019@zju.edu.cn (M.H.)

**Keywords:** stress, duration of use, flow experience, problematic SVAs use, serial mediation

## Abstract

The pervasiveness of smartphones and the popularity of short-form video applications (SVAs), such as TikTok, among middle-aged Chinese adults have raised concerns about problematic SVAs use. Although a plethora of research has examined problematic smartphone use among teenagers and young adults, scarce attention has been paid to the middle-aged group. This study integrates the psychopathological approach and the compensatory use approach to explicate problematic SVAs use among middle-aged Chinese adults. We aim to examine the relationship between stress and problematic SVAs use via the mediating roles of duration of use and flow experience. A total of 194 middle-aged adults from across the nation participated in an online survey. The results showed that stress was positively associated with problematic SVAs use. We also found that duration of use positively mediated the relationship between stress and problematic SVAs use. Furthermore, a serial mediation effect of duration of use and flow experience was found. The findings suggest that the aforementioned two approaches are complementary to each other in explicating problematic SVAs use, but the compensatory use approach explains more than the psychopathological approach does. Flow experience extends the original compensatory use approach and demonstrates the importance of incorporating techno-psychological predictors in understanding problematic SVAs use.

## 1. Introduction

Smartphones have penetrated every aspect of daily life. People use various applications on smartphones to acquire information, maintain social connectedness, and entertain themselves [1,2]. By the first half of 2021, the number of smartphone users in China reached 1007 million, accounting for 99.6% of Internet users in China [3]. In particular, by June 2021, short-form video applications (SVAs), such as TikTok, Kuaishou, and Watermelon, had attracted 888 million users across China, which was an increase of 14.4 million users compared to December 2020 [3]. SVAs enable users to create and share videos that last several seconds to a few minutes [4]. The easy-to-use interfaces, fascinating content, novel gameplay, personalized recommendation, and immersive experiences afforded by SVAs may tempt users to consume short-form videos excessively and become addicted [5,6,7]. 

This is especially the case in middle-aged adults aged between 45 and 60 years old. A report released in March 2020 indicated that middle-aged people constituted around 14.6% of new SVAs users [8], suggesting an increase in middle-aged users. Notably, some middle-aged adults demonstrated symptoms of problematic SVAs use. For instance, middle-aged users spent at least 1500 min on SVAs on a monthly basis [9]. Moreover, some of them used SVAs all day long and put SVAs use ahead of other daily routines, even if they had experienced undesirable health consequences [10,11]. An increasing number of studies have shown that problematic smartphone use may cause a range of physical and psychological problems, including low-quality sleep, body aches, dry and blurry eyes, anxiety, depression, decreased face-to-face interactions, and social isolation [12,13,14,15]. To prevent middle-aged users from suffering the health hazards associated with the problematic use of SVAs, the current study tries to explicate the mechanism of problematic SVAs use among middle-aged Chinese adults. 

Compared to the problematic use of the internet and smartphones, problematic SVAs use is a relatively new phenomenon that has received limited scholarly attention [7,14]. Notably, researchers have often used the terms excessive use, problematic use, and addiction interchangeably in internet and smartphone studies [15,16,17]. Despite the fact that these terms were measured with similar items in some cases [18,19], we suggest important differences between them. Excessive use emphasizes the large amount of time spent on using the internet or a smartphone and is often associated with potentially harmful and addiction-like symptoms [20]. Problematic use develops from excessive use, and includes the core components of addictive behaviors, such as loss of control, withdrawal, failure to reduce use, and negative consequences [20]. In comparison, addiction is a clinical disorder that results in a severe impairment in one’s physical health, psychological wellbeing, and occupational performance [21,22]. Thus, we consider problematic use an appropriate term to describe middle-aged adults’ excessive use of SVAs and the associated outcomes. According to prior research [18,23], problematic SVAs use usually involves: (1) experiencing difficulty in controlling the behavior of SVAs use; (2) manifesting a state of negative affect when SVAs usage is not possible; (3) craving SVAs usage; and (4) reporting negative physical and psychological consequences due to overuse of SVAs [24,25]. 

We employ the psychopathological approach and the compensatory use approach [26,27]—two major approaches to internet addiction and smartphone addiction—to explicate problematic SVAs use. The psychopathological approach posits that individuals experiencing psychopathologies, such as depression, anxiety, loneliness, and low self-esteem, are vulnerable to becoming addicted to the internet and smartphone applications [18,19,20,21]. Contrary to portraying smartphone overuse as compulsive and pathological, the compensatory use approach assumes that negative life situations motivate individuals to use smartphones and online applications to alleviate negative feelings, but excessive use may sometimes lead to addiction-like symptoms [17]. Despite these differing assumptions about the cause of the addiction, we try to integrate these two approaches to comprehensively understand problematic SVAs use. The reasons for the integration are as follows: (1) problematic use is usually rooted in pathological disorders [28], which entails the use of the psychopathological approach, and (2) SVAs afford users the compensatory potential to experience enjoyment and escape from negative life situations, which indicates the necessity of including the compensatory use approach. Accordingly, informed by the psychopathological approach, we treat stress—a maladaptive psychological state commonly experienced by middle-aged adults [29]—as a predictor of problematic SVAs use. Moreover, referring to the compensatory use approach, we include the duration of SVAs use and flow experience as two mediators between stress and problematic SVAs use. 

### 1.1. Stress and Problematic SVAs Use

Stress refers to the emotional or physical tension arising from sudden or long-term situations that individuals struggle to manage, especially when individuals find it difficult to meet social and environmental expectations [26,27]. Stress is manifested through several psychological indicators, such as difficulties in relaxation, nervous arousal, becoming easily upset, overreaction, and impatience [30]. Middle-aged people are often in a state of stress that peaks at around 47 or 48 years old [29]. Many high-stress situations can occur for middle-aged adults, such as rapid changes in the job market, intensification of workload, financial hardship, marital instability, demands related to parenting, and providing care for the elderly [28,31]. High levels of stress are associated with poor psychological health, triggering emotional disorders, depression, and impairment in self-esteem and decision-making ability [29,32,33].

Conceptually, the psychopathological approach to internet addiction is developed from the diathesis-stress framework, which assumes that abnormal behaviors result from the predisposed psychopathologies [26]. Prior studies have found that maladaptive psychological states (e.g., depression, anxiety, loneliness, low self-esteem, and stress) might render individuals vulnerable to the problematic use of online applications [26,34]. For instance, those suffering from depression, anxiety, and loneliness are at high risk for problematic smartphone use [19,31,35]. In a similar vein, as a person’s stress level increases, the possibility of them overusing the internet or their smartphones also increases [36,37,38,39]. This is because a stressful mental state tends to impair one’s self-control ability and self-efficacy, which makes one prone to problematic behaviors [32,38]. Thus, according to the psychopathological approach, we infer that middle-aged adults with higher levels of stress are more vulnerable to problematic SVAs use compared to those with lower levels of stress. More formally, we posit the following hypothesis:

**Hypothesis** **1** **(H1).** *Stress is positively associated with problematic SVAs use*.

### 1.2. Duration of Use as a Mediator 

Duration of SVAs use may serve as a mediator between stress and problematic SVAs use. Referring to prior research [40], we define duration of use as the total amount of time spent on SVAs daily. We draw upon the compensatory use approach to internet addiction to explicate the mediating role of duration of use.

Unlike the psychopathological approach, which views internet addiction as pathological, the compensatory use approach interprets excessive internet use as a coping strategy grounded in understandable but not always healthy motivations that may lead to addiction-like symptoms [17]. The compensatory use approach assumes that difficult life situations can motivate individuals to use online applications to escape from their difficulties [17]. Noticeably, prior empirical studies have demonstrated that individuals in stressful states used smartphones or the internet more often [38,41,42], and such usage relieved their negative feelings from stressful life situations [32,43,44]. Given that watching short-form videos can help users relax [4,6], middle-aged adults are likely to rely on SVAs to alleviate stress. Hence, the more under stress a middle-aged person is, the more likely it is that they are. to spend a longer time watching short-form videos. We posit the following hypothesis:

**Hypothesis** **2** **(H2).** *Stress is positively associated with duration of use*.

The compensatory use approach also proposes that, in addition to desirable outcomes, individuals may use the internet excessively, which sometimes develops into addiction-like symptoms [17]. Repeated and continuous use of digital devices for need satisfaction increases the risk of addiction [34]. A considerable number of studies have demonstrated that a longer duration of smartphone usage is positively related to problematic smartphone use [18,40,45,46]. Based on these findings, we posit that a similar relationship exists between the duration of SVAs use and the problematic use of SVAs:

**Hypothesis** **3** **(H3).** *Duration of use is positively associated with problematic SVAs use*.

### 1.3. Flow Experience as a Mediator 

Flow experience can be another mediator between stress and problematic SVAs use. Flow experience represents a state of enjoyment and exhilaration in which an individual is getting fully involved in an activity [38]. In such a situation, an individual takes full control of his or her actions and feels little distinction between self and environment or between past, present, and future [47,48]. Web-based technologies enable users to experience flow through feelings of temporal dissociation, focused immersion, heightened enjoyment, and curiosity [49,50,51]. In particular, the easy-to-use interface and attractive contents of SVAs are highly likely to afford users a flow experience [4,6,7] in which they become completely immersed, lose track of time, and feel heightened enjoyment and curiosity.

Research on the psychophysiology of flow experience offers some insights into understanding the association between stress and flow experience. Cortisol, a hormone that rises in response to stress, has been shown to impair flow experience [52]. Empirical evidence has demonstrated that enduring and severe stress can increase one’s cortisol level and, thus, hinder one’s flow experience [52]. In the current study, we speculate that middle-aged adults tend to experience chronic stress due to rapid changes in the job market and the increasing pressure of maintaining relationships at home [53,54]. Thus, we infer that the persistent stress in the daily lives of the middle-aged is likely to prevent them from experiencing flow:

**Hypothesis** **4** **(H4).** *Stress is negatively associated with flow experience*.

The association between flow experience and problematic SVAs use can be understood from the lens of the brain’s reward system. The brain’s reward system reinforces human behaviors related to rewards, and consists of monetary benefit and psychological gratification, which are main causes of addictive behaviors [55]. For instance, once people who are addicted to gambling experience a winning streak, they tend to believe that they can win back all their losses, which leads to further addiction [56]. Similarly, the reward of continuous wins and immediate achievement are the reasons for online gaming addiction [57,58]. Flow experience is a mental reward received during SVAs usage in which users experience an optimal state of concentration, enjoyment, and transcendence of self [48,51]. Considering that previous research has supported a positive association between flow experience and addiction to the internet or smartphones [49,59], we expect that the positive feelings associated with flow experience could potentially lead to problematic SVAs use. We posit the following hypothesis:

**Hypothesis** **5** **(H5).** *Flow experience is positively associated with problematic SVAs use*.

### 1.4. A Serial Mediation Model 

People enter a flow state when they are immersed in a task that eliminates external and internal distractions [48]. Smartphone use is a time-consuming activity that triggers users’ flow experience [60]. Both immersion and temporal dissociation—two major indicators of flow experience—are gained through continuous internet roaming [50]. The reference algorithm of SVAs platforms possesses a great understanding of users’ habits, interests, and personal experiences [4,7], and SVAs platforms constantly provide viewers with interesting content capable of triggering users’ flow experiences. In this study, we expect that SVAs users tend to achieve flow experience when they spend a longer time watching or creating short-form videos. Thus, the following hypothesis is proposed:

**Hypothesis** **6** **(H6).** *Duration of use is positively associated with flow experience*.

**Figure 1:**
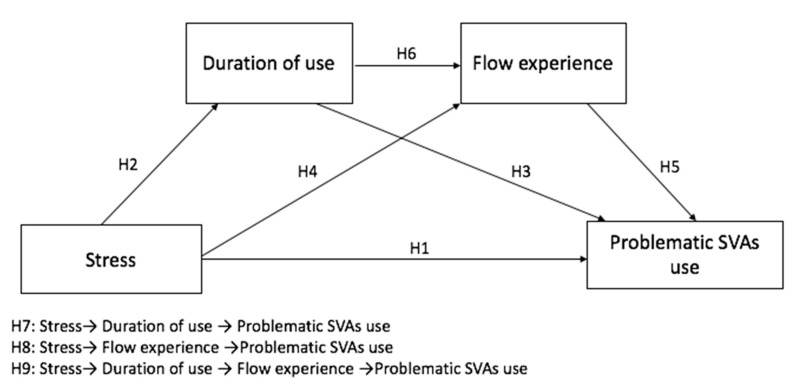
The hypothesized model.

Taken together, the above-posited hypotheses constitute a serial mediation model. Specifically, we put forward three paths to illustrate the mediating roles of duration of use and flow experience between stress and problematic SVAs use. Figure 1 presents the hypothesized model.

**Hypothesis** **7** **(H7).** *Duration of use mediates the association between stress and problematic SVAs use*.

**Hypothesis** **8** **(H8):** *Flow experience mediates the association between stress and problematic SVAs use*.

**Hypothesis** **9** **(H9).** *Duration of use and flow experience serially mediate the relationship between stress and problematic SVAs use*.

## 2. Materials and Methods

### 2.1. Participants 

We conducted a cross-sectional online survey to collect data. We used the sampling service of Sojump (http://www.sojump.com, accessed on 7 November 2021), a professional online survey website in China, to recruit participants. Sojump has 2.6 million registered respondents with diverse demographic features distributed across mainland China. This sampling strategy has been widely used to examine various social issues in China, such as air pollution, the use of renewable energy, and the development of e-commerce [61,62,63]. Our survey began on 16 October 2021, and ended on 20 October 2021. A total of 291 respondents in Sojump’s survey pool were recruited to participate in this study. The institutional review board at the authors’ affiliated university approved the data collection protocol. 

According to the widely used definition of middle-aged adults [64,65], to be eligible for our study, participants had to be between 45 and 59 years old and had to have experience using SVAs. Additionally, we considered questionnaires invalid if they met one of the two criteria: (1) made multiple submissions using the same IP address or (2) did not pass any of the four attention checks (e.g., “please select ‘strongly disagree’”). After deleting invalid cases, the final sample included 194 cases (Mage = 49.93, SD = 3.05). Table 1 presents the demographic features of these respondents.

Compared to teenagers and young adults, middle-aged SVAs users were more difficult to reach, which is why we collected a relatively small sample. Nevertheless, according to the strictest criteria of 10–20 cases per variable for data analysis [66], our sample size was acceptable (n = 194) because we had 3 indicator variables and 5 control variables. 

### 2.2. Measures 

Adapted from the prior instrument [30], stress was measured with 10 items on a 5-point Likert scale (1 = “strongly disagree”, 5 = “strongly agree”). Items included four dimensions: (1) difficulty relaxing (e.g., “I find it hard to wind down”); (2) nervous arousal (e.g., “I feel that I am using a lot of nervous energy”); (3) easily upset/agitated (e.g., “I find myself getting upset rather easily”); and (4) irritable/overreactive (e.g., “I tend to overreact to situations”). The 10 items were averaged, with higher scores suggesting higher levels of stress (M = 2.46, SD = 0.86, Cronbach’s α = 0.92).

A single item was used to measure the duration of SVAs use: “On average, how long do you use SVAs every day?” Answers were scored on a 10-point scale: (1) less than 10 min, (2) 11–20 min, (3) 21–30 min, (4) 31 min–1 h, (5) 1–1.5 h, (6) 1.5–2 h, (7) 2.5–3 h, (8) 3–4 h, (9) 4–5 h, and (10) more than 5 h (Median = 5.00, SD = 1.76).

Referring to previous instruments [49,50], we used 13 items regarding temporal dissociation, focused immersion, heightened enjoyment, and curiosity to measure flow experience. A 5-point Likert scale was used (1= “strongly disagree”, 5 = “strongly agree”). Example items included: “Sometimes I lose track of time when I am using SVAs,” “While using SVAs, I am completely absorbed,” “Using SVAs provides me with a lot of enjoyment,” and “Using SVAs excites my curiosity.” The 13 items were averaged to create an addictive index (M = 3.83, SD = 0.52, Cronbach’s α =0.83).

According to the prior measurement of excessive smartphone use, problematic smartphone use, and Internet addiction [22,49,67,68], we compiled a 13-item scale consisting of four dimensions to measure problematic SVAs use: (1) loss of control (e.g., “I will never give up using SVAs, even when my life is already greatly affected by them”), (2) withdrawal (e.g., “I feel anxious if I have not checked for SVAs updates for some time”), (3) craving (e.g., “I often have SVAs in my mind even when I am not using them”), and (4) negative life consequences (e.g., “I am often late for appointments because I am engaged with SVAs when I should not be”). Items were measured on a 5-point Likert scale (1 = “strongly disagree”, 5 = “strongly agree”). The internal consistency of the scale was satisfactory (Cronbach’s α = 0.90). The factor structure of the scale was tested using confirmatory factor analysis with AMOS version 23. The following cutoff values of model fit indices were used to assess the model structure: χ^2^/df was less than 8, the goodness of fit index (GFI) and the comparative fit index (CFI) were greater than 0.90, and the root mean square of approximation (RMSEA) and the standardized root-mean-square residual (SRMR) were less than 0.08 [69]. The factor structure of the newly compiled scale was acceptable: χ^2^/df = 2.49, GFI = 0.91, CFI = 0.92, RMSEA = 0.08, SRMR = 0.06. Additionally, the factor loadings of all the items on their related dimensions were generally high, and all exceeded the cutoff value of 0.40 [70]. Finally, a composite index was created by calculating the mean score of the 13 items, with higher values indicating a stronger tendency toward problematic SVAs use (M = 3.02, SD = 0.75).

Regarding control variables, age was measured as a continuous variable (M = 49.93, SD = 3.05) and gender as a dichotomous variable (53.0% males). Monthly income (median = 5.00, or 5001–8000 RMB, SD = 1.36) and education level (median = 6.00, or higher vocational school, SD = 1.47) were both measured as ordinal variables. Additionally, self-control was considered a control variable, due to its association with problematic smartphone use [71,72,73]. Referring to the previous instrument [74,75], we measured self-control with four items on a 5-point Likert scale (1 = “strongly disagree”, 5 = “strongly agree”): (1) “I have a hard time breaking bad habits” (reverse coded); (2) “Sometimes I cannot stop myself from doing something, even if I know it is wrong” (reverse coded); (3) “I often act without thinking through all the alternatives” (reverse coded); and (4) “I wish I had more self-discipline” (reverse coded). The four items were averaged, with higher scores suggesting higher levels of self-control (M = 2.87, SD = 0.77, Cronbach’s α = 0.74).

### 2.3. Analytical Strategy 

We first used SPSS version 26.0 to calculate the means and standard deviations of the examined variables and Pearson correlations between them. Afterwards, we conducted an OLS regression analysis using SPSS version 26.0 to compare the variance in problematic SVAs use explained by the predictors. Finally, we used PROCESS version 3.5—an SPSS macro developed by Andrew F. Hayes—to test the research hypotheses. The hypotheses constitute a serial mediation model with two mediators. Hence, we chose Model 6 in the PROCESS templates to run the statistical analysis [76]. Problematic SVAs use was entered as the outcome variable and stress as the independent variable. Duration of use and flow experience were entered as mediators. Gender, age, monthly income, education level, and self-control were entered as covariates. We tested the mediation effects with 5000 bootstrap samples at 95% confidence intervals [77]. Standardized coefficients were reported. 

## 3. Results 

### 3.1. Preliminary Results 

Table 2 presents the means, standard deviations, and bivariate correlations between the examined variables. The mean value of problematic SVAs use was 3.02 on a 5-point scale, suggesting a moderate level of the problematic use of SVAs among middle-aged Chinese adults. Stress was significantly and positively correlated with duration of use (r = 0.23, *p* < 0.01) and problematic SVAs use (r = 0.45, *p* < 0.01), but it was not correlated with flow experience (r = 0.04, *p* = 0.62). Additionally, each two variables of duration of use, flow experience, and problematic SVAs use had a positive correlation (see Table 2). Among the control variables, education level (r = –0.18, *p* < 0.05), monthly income (r = –0.15, *p* < 0.05), and self-control (r = –0.58, *p* < 0.01) were all negatively correlated with problematic SVAs use, suggesting that middle-aged adults with higher levels of education, income, and self-control were less vulnerable to the problematic use of SAVs. 

We then ran an OLS regression analysis to predict problematic SVAs use. As shown in Table 3, all predictors accounted for about 56.6% of the variance in problematic SVAs use. In addition to the control variables, stress alone explained 3.1% of the variance in the dependent variable, duration of use explained 7.7% of the variance, and flow experience explained 12.6% of the variance in the dependent variable. 

### 3.2. Primary Results 

**Figure 2:**
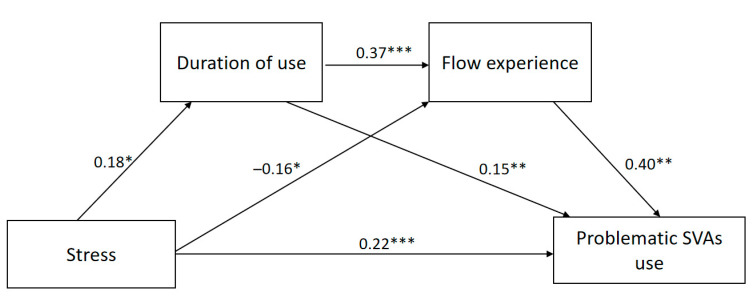
The final model based on statistical results. Note. Standardized coefficients were reported; * *p* ≤ 0.05; ** *p* < 0.01; *** *p* < 0.001.

A serial mediation analysis was performed to test the hypothesized model. The results are presented in Table 4 and Figure 2. 

Consistent with H1, stress was positively associated with problematic SVAs use (β = 0.22, *p* < 0.001). Stress was also positively correlated with duration of use (β = 0.18, *p* < 0.05), which was itself positively associated with problematic SVAs use (β = 0.15, *p* < 0.01), showing support for H2 and H3. Besides, the association between stress and flow experience was marginally significant (β = –0.16, *p* = 0.05), showing that H4 was basically supported. Supporting H5, flow experience was positively correlated with problematic SVAs use (β = 0.40, *p* < 0.001). Moreover, duration of use and flow experience were positively correlated with each other (β = 0.37, *p* < 0.001), supporting H6.

Bootstrap tests with 5000 samples were used to examine the significance of the indirect effects of stress on problematic SVAs use via duration of use and flow experience. A 95% confidence interval that did not contain zero suggested statistical significance [78]. As demonstrated in Table 5, duration of use significantly and positively mediated the association between stress and problematic SVAs use (β = 0.03, CI [0.0003, 0.06]), which supported H7. Nevertheless, the mediation effect of flow experience was not significant (β = –0.06, CI [–0.13, 0.004]), thereby rejecting H8. Furthermore, duration of use and flow experience serially mediated the relationship between stress and problematic SVAs use (β = 0.03, CI [0.002, 0.06]). Thus, H9 was supported. 

## 4. Discussion

The increasing pervasiveness of smartphones and the wide popularity of SVAs have rendered middle-aged Chinese adults vulnerable to the problematic use of SVAs. Drawing upon the psychopathological approach and the compensatory use approach, this study explored the relationship between stress and problematic SVAs use. Duration of use and flow experience were incorporated into this relationship to illuminate the underlying mechanism. When taken together, the findings of this study shed light on the psychological (i.e., stress), behavioral (i.e., duration of use), and techno-psychological (i.e., flow experience) predictors of problematic SVAs use. This not only offers theoretical insights into understanding the problematic use of SVAs, but also has practical implications for targeted prevention of and intervention in problematic SVAs use.

### 4.1. Theoretical Implications

First, the proposed model in this study integrates the psychopathological approach and the compensatory use approach, and demonstrates that these two approaches are complementary to each other in explicating the problematic use of SVAs. Compared to prior research that viewed the compensatory use approach as an alternative to the psychopathological approach [27], the integration of these two approaches provides us with a comprehensive understanding of the mechanism underlying problematic SVAs use. On the one hand, stress was positively associated with problematic SVAs use, suggesting that high levels of stress might be accompanied by impaired self-control ability, rendering individuals vulnerable to developing problematic SVAs usage. These findings are in line with the psychopathological approach, which assumes a direct link between maladaptive psychological states and problematic smartphone use [19,31,35]. On the other hand, the mediating roles of duration of use and flow experience between stress and problematic SVAs use corroborate the compensatory use approach [32,43,44]: Middle-aged adults used SVAs to escape from the stresses of daily life. Nevertheless, the excessive time spent on SVAs and the associated flow experience tended to cause the problematic use, which indicates that SVAs’ compensatory potential of escaping from the stresses might bring about negative outcomes such as SVAs overuse [17]. 

Second, our study shows that the compensatory use approach is more capable of explicating problematic SVAs use among middle-aged adults than the psychopathological approach is. Specifically, stress alone explained about 3.1% of the variance in problematic SVAs use, whereas duration of use and flow experience together explained 20.3% of the variance in the dependent variable. The following reasons might explain this difference. The mean value of problematic SVAs use was 3.02 on a 5-point scale, suggesting a moderate level of the problematic use. Because severe problematic use and addiction are usually associated with psychopathologies [26], the psychopathological approach thus has limited explanatory power in explicating the moderate level of problematic SVAs use seen in this study. In comparison, SVAs primarily allow users to continuously watch videos and become absorbed in video contents [4,7], thereby realizing the compensatory potential of forgetting stressful life situations, as the compensatory use approach suggests. 

Third, by incorporating flow experience, our model extends the original compensatory use approach, which focused on the psychological mechanisms of the problematic use of the internet and smartphones, such as motivations [17]. Flow experience—a techno-psychological predictor—highlighted the importance of including certain mental states afforded by certain technologies in understanding the problematic use of these technologies. The auto-loop displays of short-forms videos, the unpredictability of creative content in the next video, and the chance of interacting with content publishers all lead to non-stop watching. Because flow experience, which is obtained through such use of SVAs, functioned as a mental reward that tempted middle-aged users to watch short-form videos, there is a risk of this use developing into problematic use. Notably, when compared to duration of use (R2 = 7.7%), flow experience (R2 = 12.6%) explained more variance in problematic SVAs use, emphasizing the importance of incorporating techno-psychological predictors into the compensatory use approach. Compared to the majority of related research, which has examined problematic smartphone use in general [40,49,67], the extended compensatory use approach encourages researchers to focus on specific smartphone applications and explore the role of the associated techno-psychological predictors in explaining users’ problematic use of these applications. 

Fourth, the mediating role of duration of use is line with previous research, in which the excessive use of online applications was often associated with the problematic use of these applications [18,40,45,46]. This indicates that duration of use is a necessary but not sufficient predictor of problematic SVAs usage. In other words, middle-aged adults would not develop problematic SVAs use without spending an excessive amount of time on SVAs; however, a longer duration of use does not necessarily cause the problematic use. This echoes the serial mediation effect in which duration of use influenced problematic SVAs use mainly through flow experience. 

Finally, the mediating role of flow experience between stress and problematic SVAs use was not significant. The marginally significant correlation between stress and flow experience (β = –0.16, *p* = 0.05) contributed to the nonsignificant indirect effect of stress on problematic SVAs usage through flow experience. Moreover, the complicated relationship between stress and flow experience might explain the insignificant indirect effect. Prior research has suggested that the relationship between stress and flow experience might be u-shaped [79]; namely, moderate levels of stress could facilitate flow experience, whereas severe and chronic stress could hinder flow experience [52]. Thus, we advise future research to use experimental design to examine the possible curvilinear relationship between stress and flow experience. This endeavor could offer researchers a better understanding of the role of flow experience between stress and problematic SVAs use. 

### 4.2. Practical Implications

The proposed model in the current study has several implications for properly managing problematic SVAs use among middle-aged adults. For middle-aged adults, the use of SVAs can be a double-edged sword: rational use can alleviate stress, but overuse can cause addiction-like symptoms. Given the relative prominence of the compensatory use approach in explicating problematic SVAs use, we first advise SVAs platforms to set up protective modes to prevent middle-aged users from excessive usage. Only when the duration of SVAs use is properly controlled can the tendency of problematic SVAs use resulting from a long duration of use and the associated flow experience be reduced. Additionally, the psychopathological approach advises middle-aged adults to participate in leisure activities that could ease their stress, thereby lowering the likelihood of developing the problematic use of SVAs. 

### 4.3. Limitations and Future Research 

This study has several limitations. First, we collected data only from China. Given that SVAs usage has become prevalent in many countries across the world [80,81], the one-country-based design has limited capacity to understand this global phenomenon. Accordingly, in the future, researchers could conduct comparative research regarding the problematic use of SVAs between different countries. Second, due to the cross-sectional nature of the survey data, we could not claim causality between the examined variables. Thus, we suggest researchers use a longitudinal design to collect data (for instance, [67], thereby testing the causal effects hypothesized in the model. Third, the quantitative nature of the current study makes it difficult for us to discuss the meanings embedded in each of the examined variables and in the relationships between them. Future research could use qualitative methods to explore these nuanced meanings. Fourth, the self-reported measures of stress and problematic SVAs use were prone to social desirability and estimation biases [40]. Similarly, the self-assessment of the duration of SVAs use might suffer from recall biases [82]. Future research could use objective data, such as the recorded time of SVAs use on smartphones, to increase reliability and validity. Fifth, despite the fact that our sample size (n = 194) was adequate given the estimated model, future studies are advised to recruit more middle-aged respondents to further validate the model. 

## 5. Conclusions

This is one of few studies examining problematic SVAs use among middle-aged Chinese adults, who have received scarce scholarly attention compared to teenagers or young adults [16,17,24]. With duration of use and flow experience incorporated as two mediators between stress and problematic SVAs use, our study contributes to the extant research in several ways. First, we demonstrate the validity of the psychopathological approach [26,34] and the compensatory use approach [27] in explicating problematic SVAs use. Second, the findings indicate that these two approaches are complementary to each other in explaining problematic SVAs use, updating the prior argument that considered the compensatory use approach only as an alternative to the psychopathological approach [27]. Third, our model shows that the compensatory use approach may be more explanatory than the psychopathological approach in understanding the problematic use of SVAs. Last but not least, flow experience extends the original compensatory use approach, which mainly focused on motivations [17], and highlights the importance of including techno-psychological predictors in accounting for problematic SVAs use.

## Figures and Tables

**Table 1:** Demographic characteristics of the participants.

Measure	Item	Frequency	Percentage(%)
Gender	Male	103	53.0%
	Female	91	46.9%
Area	Beijing, Shanghai, Tianjin, Chongqing, Shenzhen	43	22.1%
	Capital city of province	59	30.4%
	Prefecture-level cities	49	25.2%
	Counties and towns	39	20.1%
	Administrative villages	4	2.0%
Monthly income	Less than RMB 1500	3	1.5%
	RMB 1501–2000	4	2.0%
	RMB 2001–3000	11	5.6%
	RMB 3001–5000	38	19.5%
	RMB 5001–8000	75	38.6%
	RMB 8001–12,000	33	17.0%
	RMB 12,001–20,000	22	11.3%
	More than RMB 20,000	8	4.1%
Education level	Never attend to school	0	0%
	Primary school	6	3.0%
	Middle school	15	7.7%
	High school	30	15.4%
	Vocational high school	16	8.2%
	Higher vocational school	55	28.3%
	Bachelor	70	36.0%
	Master	2	1.0%
	PhD	0	0%

**Table 2:** Means, standard deviations, and bivariate correlations between examined variables.

Variables	M	SD	1	2	3	4	5	6	7	8	9
1. Stress	2.46	0.86	-								
2. Duration	5.25	1.76	0.23 **	-							
3. Flow experience	3.83	0.52	0.04	0.40 **	-						
4. Problematic SVAs use	3.02	0.75	0.45 **	0.43 **	0.52 **	-					
5. Gender	1.47	0.50	0.07	0.12	0.10	0.09	-				
6. Age	49.93	3.05	−0.08	−0.03	−0.07	0.09	−0.06	-			
7. Education level	5.63	1.47	−0.15 *	−0.13	−0.12	−0.18 *	−0.06	0.04	-		
8. Monthly Income	5.09	1.36	−0.25 **	0.02	0.03	−0.16 *	−0.13	−0.04	0.54 **	-	
9. Self-control	2.87	0.77	−0.55 **	−0.24 **	−0.21 **	−0.58 **	−0.20 **	0.06	0.31 **	0.28 **	-

Note. Gender 1 = male, 2 = female; * *p* < 0.05; ** *p* < 0.01.

**Table 3:** OLS regression predicting problematic SVAs use.

Independent Variables	Problematic SVAs Use			
Block 1: Control variables				
Gender (male = 1, female = 2)	–0.02	–0.01	–0.05	–0.05
Age	0.12 *	0.14 *	0.13 *	0.15 **
Education level	–0.002	–0.02	0.02	0.05
Monthly income	0.01	0.04	–0.02	–0.05
Self-control	–0.59 ***	–0.47 ***	–0.44 ***	–0.36 ***
Adjusted *R*^2^	33.0%			
Block2				
Stress		0.22 **	0.16 *	0.22 ***
Incremental adjusted *R*^2^		3.1%		
Block3				
Duration of use			0.30 ***	0.15 **
Incremental adjusted *R*^2^			7.7%	
Block4				
Flow experience				0.40 ***
Incremental adjusted *R*^2^				12.6%
Total adjusted *R*^2^	33.0%	35.9%	43.6%	56.5%

Note. Standardized coefficients were reported; * *p* < 0.05; ** *p* < 0.01; *** *p* < 0.001.

**Table 4:** Regression results for the serial mediation model.

Predictors	Duration of use	Flow Experience	Problematic SVAs Use
	β	β	β
Gender	0.10	0.03	–0.05
Age	0.01	–0.05	0.15 **
Education level	–0.16	–0.08	0.05
Monthly income	0.21 *	0.08	–0.05
Self-control	–0.13	–0.20 *	–0.36 ***
Stress	0.18 *	–0.16 *	0.22 ***
Duration of use	—	0.37 ***	0.15 **
Flow experience	—	—	0.40 ***
F	(6, 187) = 3.86	(7, 186) = 6.86	(8, 185) = 32.30
*R* ^2^	11.0%	20.5%	58.3%

Note: Gender 1 = male, 2 = female; * *p* ≤ 0.05; ** *p* < 0.01; *** *p* < 0.001.

**Table 5:** Bootstrap tests on the indirect effects.

Paths	Standardized (β)	95% Cl	
		Low	High
Stress→Duration of use→Problematic SVAs use	0.03	0.0003	0.06
Stress→Flow experience→Problematic SVAs use	–0.06	–0.13	0.004
Stress→Duration of use→Flow experience→Problematic SVAs use	0.03	0.002	0.06

## Data Availability

Please email the corresponding author, Hongliang Chen, for the data: hongliangchen@zju.edu.cn.
